# Designing and Exploration of the Biological Potentials of Novel Centrosymmetric Heteroleptic Copper(II) Carboxylates

**DOI:** 10.3390/ph16101462

**Published:** 2023-10-14

**Authors:** Viola, Niaz Muhammad, Awal Noor, Muhammad Sirajuddin, Maciej Kubicki, Shahnaz Rahim, Abdus Samad, Shaukat Shujah, Abdul Wadood, Saqib Ali

**Affiliations:** 1 Department of Chemistry, Abdul Wali Khan University Mardan, Mardan 23200, Pakistan; violamadan07@yahoo.com (V.); srahim639@gmail.com (S.R.); 2 Department of Basic Sciences, Preparatory Year Deanship, King Faisal University, Al-Hassa 31982, Saudi Arabia; 3 Department of Chemistry, University of Science and Technology Bannu, Bannu 28100, Pakistan; m.siraj09@gmail.com; 4 Faculty of Chemistry, Adam Mickiewicz University, Uniwersytetu Poznanskiego 8, 61-614 Poznań, Poland; maciej.kubicki@amu.edu.pl; 5 Department of Biochemistry, Abdul Wali Khan University Mardan, Mardan 23200, Pakistan; sabdus591@gmail.com (A.S.); awadood@awkum.edu.pk (A.W.); 6 Department of Chemistry, Kohat University of Science & Technology, Kohat 26000, Pakistan; shaukat.shujah@yahoo.com; 7 Department of Chemistry, Quaid-I-Azam University Islamabad, Islamabad 45320, Pakistan; drsa54@yahoo.com

**Keywords:** copper(II) carboxylate, paddlewheel structure, antioxidant, α-amylase inhibition, DNA interaction, molecular docking

## Abstract

Copper(II) complexes with a general formula [Cu_2_(3,4-F_2_C_6_H_3_CH_2_COO)_4_(L)_2_], where L = 2-methylpyridine (**1**) and 3-methylpyridine (**2**), are reported here. The FTIR spectra of the complexes confirmed the bridging bidentate coordination mode of the carboxylate ligand. The low (475 and 449 cm^−1^) and strong (727 & 725 cm^−1^) intensity bands in the FTIR spectra, due to Cu-N stretches and pyridyl ring vibrations, confirmed coordination of the 2-/3-methyl pyridine co-ligands in complexes **1** and **2**, respectively. A binuclear paddlewheel structural arrangement with a square pyramidal geometry was confirmed for copper atoms in the complexes via single-crystal X-ray analysis. The DPPH, ^•^OH radical, and α-amylase enzyme inhibition assays showed higher activities for the complexes than for the free ligand acid. The binding constant (K_b_ = 1.32 × 10^5^ for **1** and 5.33 × 10^5^ for **2**) calculated via UV-VIS absorption measurements and docking scores (−6.59 for **1** and −7.43 for **2**) calculated via molecular docking showed higher SS-DNA binding potential for **2** compared to **1**. Viscosity measurement also reflected higher DNA binding ability for **2** than **1**. Both complexes **1** and **2** (docking scores of −7.43 and −6.95, respectively) were found to be more active inhibitors than the free ligand acid (docking score of −5.5159) against the target α-amylase protein. This in silico study has shown that the herein reported compounds follow the rules of drug-likeness and exhibit good potential for bioavailability.

## 1. Introduction

Heteroleptic paddlewheel binuclear copper(II) carboxylates containing monodentate N-donor co-ligands constitute an important class of compounds with potential applications in bioinorganic chemistry. This class of compounds can be represented by the general formula [Cu_2_(OOCR)_4_L_2_], where four bridging carboxylate ligands form the base of the paddlewheel structure and the monodentate N-donor ligands occupy the apical positions. The two carboxylate-bridged copper(II) ions have square pyramidal geometry, and so are sterically less saturated, a structural feature of the complex that could be exploited for the interactions of the metal center with biomolecular receptors in biological activity [1]. Apart from the possible interaction of metal center, the biological properties of these com-plexes could also be tuned up to varying extent by brining small structural changes in the attached carboxylate or N-donor ligands [2,3,4,5,6,7,8,9,10,11].

According to earlier reports, the attachment of N-donor ligands to Cu(II) ions enhances the planarity in the structure and reduces the maximum attainable geometry around the Cu(II) ions [12,13,14,15]. In terms of biological activity, dinuclear complexes have shown greater activity than mononuclear complexes. Thus, the combination of carboxylates with less steric bulk and N-donor aromatic ligands provides an ideal platform for exploring their biological applications [16].

As a continuation of our previous work on copper(II) carboxylates [17], in this study, a simple synthetic route was adopted to introduce additional N-donor ligands to isolate complexes, consisting of 3,4-difluorophenylacetate and 2-/3-methylpyridine. The in vitro antioxidant and alpha-amylase inhibition potential of the synthesized complexes were evaluated. Since DNA is an important biological target of many anticancer and antiviral therapies, these compounds were screened for their DNA-binding ability using UV-visible spectroscopy and viscometry. A molecular docking study of the synthesized complexes was also performed to gain insight into the complex-DNA and complex-alpha-amylase interaction potential.

## 2. Results and Discussion

### 2.1. Synthesis

**Scheme 1:**
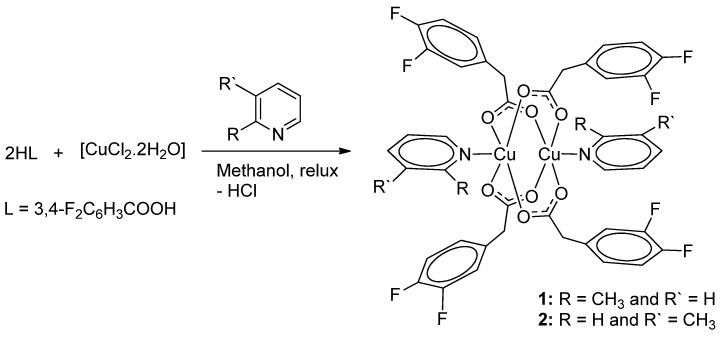
Synthetic route of the syntheses of complexes **1** and **2**.

The copper(II) complexes (**1** and **2**) were synthesized by reacting copper(II) chloride dihydrate with 3,4-difluorophenylacetic acid in the presence of either 2-methylpyridine (**1**) or 3-methylpyridine (**2**) as co-ligands in methanol under reflux condition. Sea green color complexes, soluble in methanol and DMSO, were obtained in moderate yields (Scheme 1).

### 2.2. FTIR Spectra

For the preliminary solid-state structural characterization, the FT-IR spectra of the synthesized complexes **1** and **2** were compared with that of the free ligand acid spectrum. Significant evidence confirming the complexes’ synthesis was provided by this comparison. The most explicit evidence was the absence of a broad band at 3400–2400 cm^−1^ in the complexes’ spectra. This broad band is typical for free carboxylic acid, showing OH bond vibrations [17]. During complexation, deprotonation of the carboxylate ligand occurs, so this band disappears in the complexes. Also, low intensity bands, due to Cu-O bond vibrations, appeared at 418 and 432 cm^−1^ for complexes **1** and **2**, respectively. The bands due to C=O and C-O bond vibrations at 1708 and 1279 cm^−1^, respectively, of the free ligand acid were absent in the spectra of the complexes. Instead, these bands were replaced by strong intensity bands at 1624, 1615 and 1397, 1396 cm^−1^, due to COO_asy_ and COO_sym_ vibrations in complexes **1** and **2**, respectively, indicating the bridging bidentate coordination of the carboxylate ligand to the copper center [17]. The coordination of nitrogen-donor ligands was evidenced by low intensity bands at 475 and 449 cm^−1^ in the spectra of complexes **1** and **2**, respectively [18]. Strong bands due to pyridyl ring vibrations were observed at 727 and 725 cm^−1^ in complexes **1** and **2**, respectively [19]. Complexes **1** and **2** spectra also showed strong bands due to Ar-F bond vibrations at 1208 cm^−1^. The structural findings of the IR spectra were also confirmed by single-crystal X-ray diffraction analysis of complexes **1** and **2**.

### 2.3. Crystal Structure Description of the Complexes

**Figure 1:**
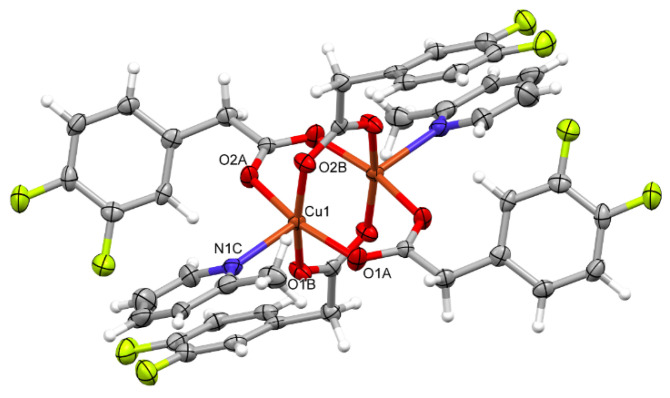
Perspective view of complex **1**; ellipsoids are drawn at the 50% probability level; hydrogen atoms are shown as spheres of arbitrary radii. The unlabeled part is related to the labeled one by the symmetry operation 1-x, 1-y, 1-z.

**Figure 2:**
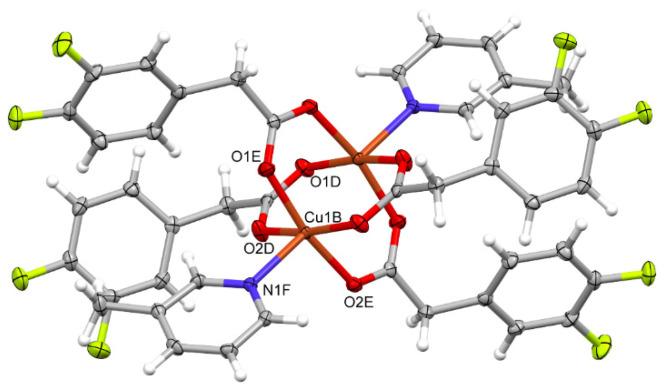
Perspective view of one of the symmetry-independent molecules of complex **2**; ellipsoids are drawn at the 50% probability level; hydrogen atoms are shown as spheres of arbitrary radii. The unlabeled part is related to the labeled one by the symmetry operation -x, -y, 2-z.

The relevant crystallographic data for complexes **1** and **2** along with the details of structure refinement are listed in Table 1. Perspective views of complexes **1** and **2** are shown in Figure 1 and Figure 2, respectively. The complexes are both C_i_-symmetrical complexes, as they lie across the inversion centers in the appropriate space groups. The asymmetric part of complex **2**’s structure actually contains two symmetry-independent halves across two different inversion centers, at 1,1/2,0 and 0,0,1. The tendency towards such symmetry is common for similar compounds. In the CSD, there are 712 fragments with bis(μ_2_-benzoato-O, O`)copper(II) complexes with N atoms at the fifth coordinating position, and among these, 612 fragments contain symmetrical molecules.

In both the complexes, Cu(II) ions are five coordinated (O_4_N), exhibit square pyramidal geometry, with four equatorial oxygen atoms belonging to the carboxylate group of four bridging 3,4-difluorobenzoate anions (Cu1–O_eq_ distances in the range of 1.958(3)–1.971(3) Å in **1** and 1.9595(17)–1.9828(17) Å in **2** (Table 2)) and the pyridine nitrogen atom of the methylpyridine. The methyl groups of the two pyridine ligands are pointing opposite to each other in square planar geometry, to minimize any steric strain. The elongated Cu1–N1 bond distance of 2.210(3) Å in **1** compared to 2.139 Å (average) in **2** could be attributed to the steric crowding of 2-methylpyridine in close proximity to the Cu center. The same could be argued for the longer Cu···Cu separation [2.6775(9) Å] in **1** than [2.6333(6) Å (average)] in **2,** but well within the range known for such compounds [17]. Table 2 lists all the relevant geometrical parameters. In general, this geometry is typical for similar compounds.

Hirshfeld surface analyses were performed to quantify and visualize the closed intermolecular atomic contacts in the crystal structures of **1** and **2**, and visualization of the Hirshfeld three-dimensional d_norm_ surfaces of the title compounds are shown in Figures S1 and S2, respectively. The intensive red spots on the surfaces, colored according to d_norm_, are related to interactions involving fluorine and oxygen atoms corresponding to the H–bonds. Figures S3 and S4 show the fingerprint plots of complexes **1** and **2**, respectively, indicating significant contributions due to different intermolecular interactions. For both compounds, the highest contributions of total Hirshfeld surfaces are attributed to H⋯F/F⋯H hydrogen bonding (30.5% (**1**) and 29.9% (**2**)), H…H (27.4% (**1**) and 26.0% (**2**)), and C…H/H…C (24.7% (**1**) and 17.0% (**2**)) π interactions. The corresponding acceptor and donor atoms showing C—H⋯O hydrogen bonds (8.6% (**1**) and 12.7% (**2**)), shown as bright red spots on the Hirshfeld surface, are the other prominent contributions. The contributions due to C…F/F…C and C…C interactions are more dominant in **2** than in **1**.

### 2.4. Antioxidant Activities

DPPH and hydroxyl (•OH) radical scavenging assays based on spectroscopic measurements are simple in vitro methods used to evaluate the antioxidant potential of test compounds. The promising results of these assays could be exploited as preliminary tests in search of drugs with potential anticancer, anti-aging, and anti-inflammatory activities [20]. Within this context, the abilities of the free ligand acid (**HL**) and synthesized complexes (**1** and **2**) to scavenge DPPH and hydroxyl radicals were determined in order to evaluate their potential antioxidant ability compared to that of vitamin C as a reference compound.

The dose-dependent (15–240 µM) responses and IC_50_ values of the compounds compared to those of standard vitamin C are shown in Table 3 and Table 4 for DPPH and hydroxyl (•OH) radical scavenging assays, respectively. The studied radicals with a single unpaired electron each are capable of accepting an electron, and thus not only lead to a decrease in the UV absorption at 517 nm, but also the color changes from violet to yellow. A decrease in absorption generally occurs when it accepts a hydrogen atom or electron from an antioxidant, to form a stable molecule. With an increase in the sample’s concentration, the radicle scavenging rates of the test compounds increase subsequently. The complexes showed significantly higher free radical scavenging ability compared to that of the free ligand acid in both the assays. This reflects the importance of the redox active copper center as a free radical scavenger. The presence of an unpaired electron in copper(II) ion could be a possible reason for the enhanced ability of the complexes to arrest the free radicals. The two complexes, despite their very close structural/composition resemblance, showed significantly different activities. Complex **1** showed higher activity compared to that of complex **2**. The difference in the antioxidant activity of the two complexes reflects the importance of the functional group position on the nitrogen-donor ligand. A slight change in the position of the functional group has a pronounced impact on the free radical scavenging ability of the complex. In complex **1**, the 2-methyl pyridine ligand has better electron donating ability than the 3-methyl pyridine of complex **2**, thus making the copper an electron-rich center for interactions with the free radicals. Complex **1** was more effective against DPPH compared to •OH radical, while complex **2** showed better efficiency against •OH than DPPH. This radical scavenging selectivity of the complexes is not unexpected, as many complexes are reported in the literature with a somewhat similar behavior against different free radicals [21].

### 2.5. Alpha-Amylase Inhibition Assay

The α-amylase catalyzed hydrolysis of carbohydrate results in elevated blood sugar levels, especially after meals, and can lead to hyperglycemia [22]. Therefore, inhibition of this enzyme is considered to be an effective therapeutic approach to control hyperglycemia [23]. In the current study, the inhibition effects of the free ligand acid and synthesized complexes on the α-amylase enzyme were investigated and compared to acrabose as a standard (Table 5). A dose-dependent enzyme inhibition response was noticed for the test compounds with the following activity order: acrabose > **1** > **2** > **HL**. In this study, the free ligand acid was the weakest inhibitor of α-amylase (IC_50_ value of 728.17 µg/mL) compared to acrabose (IC_50_ value of 207.72 µg/mL). Coordination of the deprotonated carboxylate ligand to the copper center sufficiently improved the α-amylase inhibition potential of the synthesized complexes (IC_50_ values, 437.73 µg/mL (**1**) and 455.32 (**2**) µg/mL). However, no significant activity differences were observed in the case of synthesized complexes. The enhanced activity of the metal complexes compared to the free ligand acid could be a consequence of the copper(II) ion’s ability to interact with the donor atoms present in the enzyme [24].

### 2.6. DNA Binding Study

#### 2.6.1. DNA Binding Study by Absorption Spectroscopy

UV-visible absorption measurements can be used to monitor changes occurring in λ_max_ values (red or blue shifts) and absorption intensity (hyper- or hypochromism) of either a test compound or DNA as a result of any possible interaction. These changes can be used to find some details about the mode of interaction of metal complexes with DNA [25]. In the present study, absorption titrations were made with a fixed concentration (200 μM) of complexes against varying concentrations (5–30 μM) of SS-DNA.

**Figure 3:**
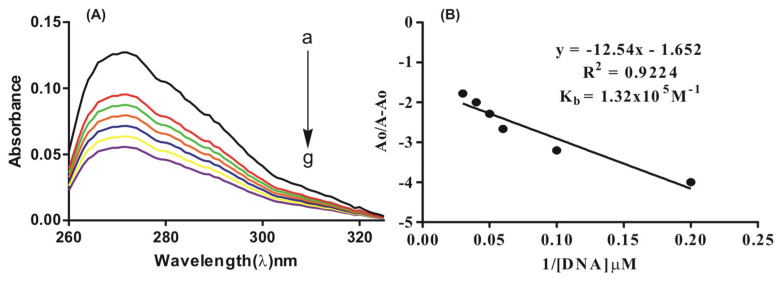
(**A**) Absorption spectrum of complex **1** in the absence (a) and presence of 5 μM (b), 10 μM (c), 15 μM (d), 20 μM (e), 25 μM (f), and 30 μM (g) DNA (the arrow direction indicates increasing concentrations of DNA); (**B**) plot of A_o_/(A-A_o_) vs. 1/[DNA] for binding constant determination of complex 1-DNA adduct.

**Figure 4:**
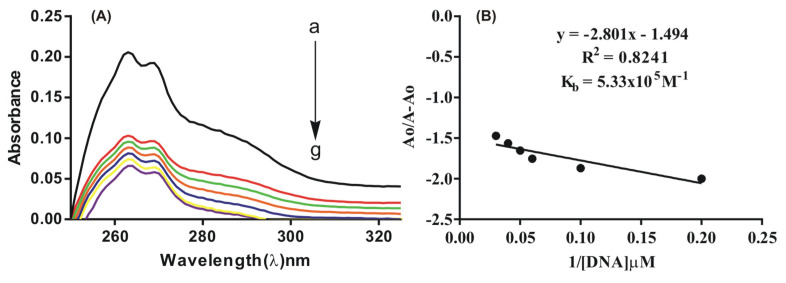
(**A**) Absorption spectrum of complex **2** in the absence (a) and presence of 5 μM (b), 10 μM (c), 15 μM (d), 20 μM (e), 25 μM (f), and 30 μM (g) DNA (the arrow direction indicates increasing concentrations of DNA); (**B**) plot of A_o_/(A-A_o_) vs. 1/[DNA] for binding constant determination of complex 2-DNA adduct.

The synthesized complexes showed pronounced hypochromism with a very small red shift of about 1 nm upon successive addition of SS-DNA, as shown in Figure 3A and Figure 4A for complexes **1** and **2**, respectively. This behavior suggests a mixed binding mode of the complexes with DNA, consisting of partial intercalation of the complexes into the base pairs of the DNA, along with groove binding [19,25].

The extent of complex-DNA binding was assessed by calculating intrinsic binding constant (K_b_) values using the well-known Benesi–Hildebrand equation [26].

$$\frac{A_{o}}{A-A_{0}}=\frac{\varepsilon_{G}}{\varepsilon_{H\text{-}G}-\varepsilon_{G}}+\frac{\varepsilon_{G}}{\varepsilon_{H\text{-}G}-\varepsilon_{G}}+\frac{1}{K_{b}[DNA]}$$

where K_b_ is the binding constant, A is the absorbance of complex-DNA adduct, A_o_ is the absorbance of pure complex solution, [DNA] is the concentration of SS-DNA in mol/L, ε_H-G_ is the molar absorption co-efficient of each complex-DNA adduct, and ε_G_ is the molar absorption co-efficient of pure complex. The K_b_ value was calculated from the intercept to slope ratio of the plot of 1/[DNA] along the abscissa vs. A_o_/A−A_o_ along the ordinate, as shown in Figure 3B and Figure 4B for complexes **1** and **2**, respectively.

The calculated K_b_ values were found to be 1.32 × 10^5^ and 5.33 × 10^5^ for complexes **1** and **2**, respectively. The K_b_ values show significant interaction of the synthesized complexes with the SS-DNA compared to the previously reported structurally related paddlewheel copper(II) complexes [17,27]. The difference in K_b_ values of the complexes reflects the importance of the functional group position on the same ligand. The position of a functional group on a coordinated ligand can significantly affect the relative orientation of a complex during its interaction with a biomolecule that can affect the bioactivity of the metal complexes. The calculated ΔG values of −29.20 and −32.67 KJ/mol for the complexes **1** and **2**, respectively, reflect the spontaneity of the complex-DNA interaction.

#### 2.6.2. DNA-Binding Study by Viscometry

**Figure 5:**
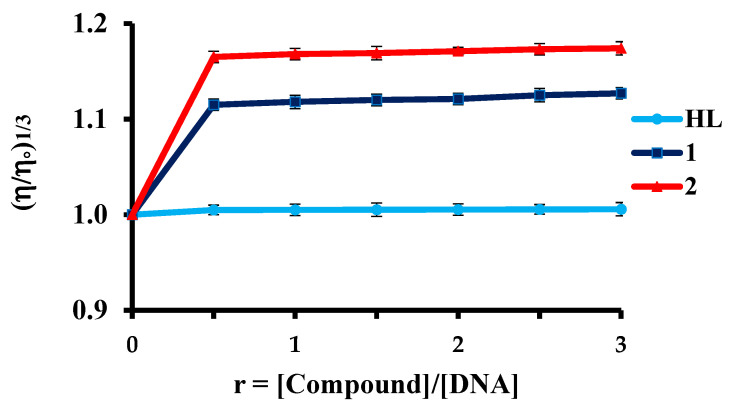
Effects of increasing the amounts of free ligand acid **HL** and the complexes (**1** and **2**) on the relative viscosity of SS-DNA.

The interaction mode of the compounds with DNA was further investigated via viscometry, and the results are shown in Figure 5. The free ligand acid has no effect on the relative viscosity of the DNA. However, the synthesized complexes significantly increased the relative viscosity of the DNA. Complex **2** increased the viscosity of DNA more than complex **1**. The significant increase in the relative viscosity of DNA in the presence of complexes compared to that in the free ligand acid reflects the importance of metal in DNA interactions. Changes in viscosity are sensitive towards length changes in DNA molecules. In the case of the intercalation mode of action, a DNA molecule lengthens to adjust the binding complex and as a result the viscosity of the complex-DNA adduct increases [28]. In groove binding, a steady increase in the relative viscosity of DNA occurs with an increasing concentration of complex [28]. A pronounced increase in the relative viscosity of DNA upon addition of complexes **1** and **2** followed by a slight increase in complex-DNA adduct viscosity show intercalation and groove binding as the possible mode of complex-DNA interactions.

### 2.7. Molecular Docking Studies

#### 2.7.1. Docking of Complexes with Alpha-Amylase

**Figure 6:**
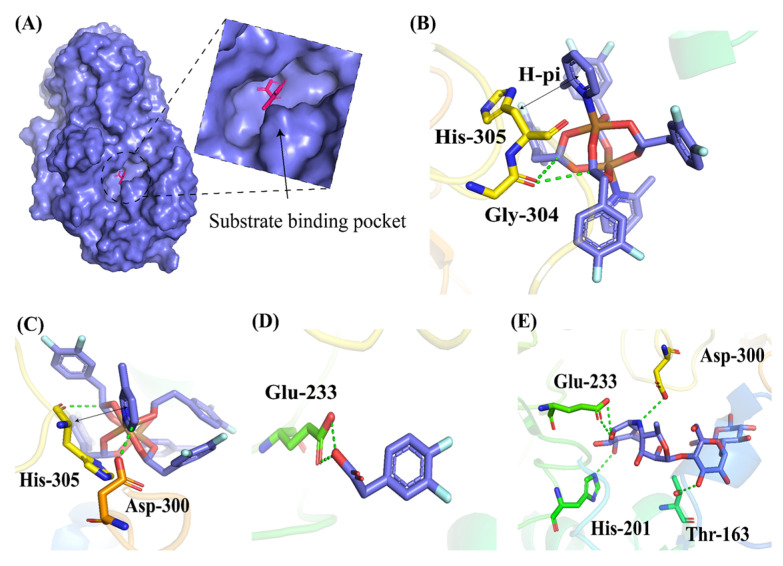
Docked conformations of the **HL**, the compleses **1** and **2**, and the control: (**A**) The surface representation of the substrate binding pocket of alpha-amylase; (**B**) the 3D binding mode of **1,** (**C**) **2**, (**D**) **HL**, and (**E**) control (acarbose), against alpha-amylase.

The molecular docking studies of the synthesized complexes showed a wide range of potential interactions with the substrate-binding site (Figure 6A) of alpha-amylase. In order to assess the accuracy of the MOE docking, the co-crystallized ligand (PDB ID 3BAJ) was extracted out of the active site and docked once again into the binding site of alpha-amylase. Our procedure was quite accurate, as shown by the RMSD value of 1.0214 between the top-ranked docked conformation and the co-crystallized ligand [29] (Figure S8). The binding site residues involved in the interactions with the complexes and control compound included Thr 163, His 201, Glu 233, Asp 300, Gly 304, and His 305. The interaction details and docking scores are listed in Table 6. The control, i.e., acarbose, (docking score of −8.73) established significant interactions with the catalytic site residues of amylase. Thr 163, Glu 233, and Asp 300 developed hydrogen bond donor interactions, while His 201 showed one hydrogen bond acceptor interaction with acarbose, as shown in Figure 6E. Complex **1** (docking score of −7.43) formed two hydrogen bond donor interactions and one C-H–pi interaction with Gly 304 and His 305 amino acids residues of the target protein, as shown in Figure 6B. Complex **2** (docking score of −6.95) formed two hydrogen bond donor interactions with His 305 and Asp 356. His 305 was also found in the C-H–pi interaction with complex **2**, as shown in Figure 6C. The free ligand acid was found to be the least active compound with a docking score of −5.5159 and having developed two H bond donor interactions with Glu 233 active residues of the target protein, as shown in Figure 6D.

#### 2.7.2. Docking of Complexes with Salmon Sperm DNA

**Figure 7:**
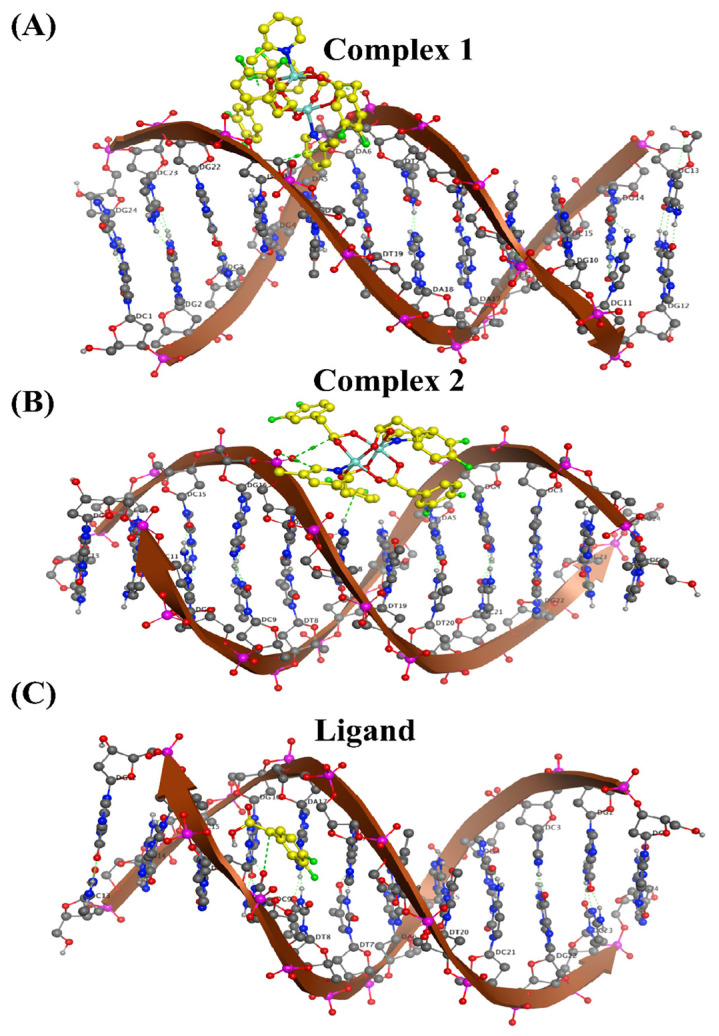
3D representation of the binding poses of: (**A**) Complex **1;** (**B**) complex **2;** (**C**) free ligand acid **HL**, with SS-DNA.

The interaction of the synthesized compounds with SS-DNA was further confirmed theoretically by a molecular docking analysis in order to gain insight about the mechanism of interactions. The MOE’s rigid molecular docking approach was utilized to evaluate the binding modes of the synthetic complexes with SS-DNA duplex. The whole DNA duplex was considered for docking to determine the preferred binding sites, binding strength, and the orientation of complexes **1** and **2** within the DNA groove and base pairs. The interaction details of both the complexes (**1** and **2**) and the free ligand acid are presented in Table 7. The docking analysis reveals that the complexes electrostatically interact with the oxygen atom on the phosphate backbone of DNA outer edge stacking. The synthesized complexes form hydrogen bond donors and C-H–pi interactions in contact with the DNA functional groups that define the minor groove because they fit snugly into the curved contour of the target DNA in the groove. The theoretical interaction analysis of the complexes strongly validated the experimental finding of the spectroscopic analysis of the complexes. Complex **2**, with a docking score of −7.43, forms two hydrogen bond donor interactions with DA17 and one C-H–pi interaction with DA18, as shown in Figure 7B. The C-H–pi interaction is a type of interaction that involves the π system. This type of bond is formed between a hydrogen atom and the π cloud. This type of interaction exists where aliphatic or aromatic CH groups are involved. Complex **1** (docking score of −6.59) shares one hydrogen bond donor and one C-H–pi interaction with DT 7 and DC 21, respectively, as shown in Figure 7A. The free ligand acid, with a docking score of −5.11, forms one H-donor interaction with DC 9, as shown in Figure 7C.

### 2.8. In Silico Studies

Table 8 presents the physicochemical parameters including solubility, lipophilicity, toxicity risks, drug-likeness, pharmacokinetics and medicinal chemistry of the synthesized compounds, evaluated according to the following filters: Lipinski [30], Ghose [31], Veber [32], Egan [33] and Muegge [34] using the SwissADME server [35]. An orally-active compound should follow these filters and should not have more than one violation. Since both complexes 1 and 2 have the same molecular weight and formula, their SwissADME data is the same.

**Figure 8:**
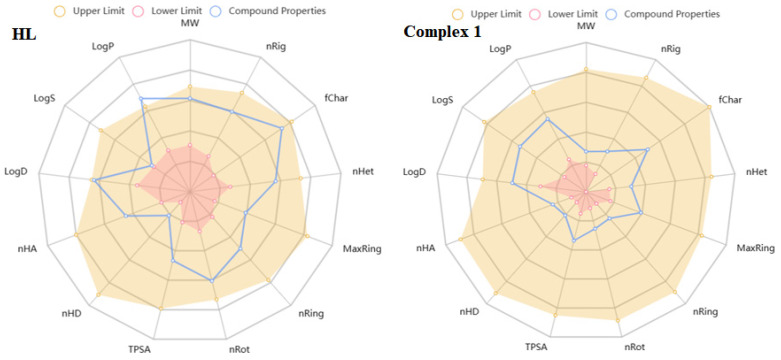
Bioavailability radar mage of **HL** and complex **1**.

The molecular weight range was 172.13−498.91 g/mol (asymmetric unit in the case of **1** and **2**), and the numbers of hydrogen bond donors and hydrogen bond acceptors to/from the H_2_O molecules in an aqueous solution were 1 (**HL**) and 0 (complexes **1** and **2**) and 4 (**HL**) and 8 (complexes **1** and **2**), respectively. The predicted numbers of rotatable bonds were in the range of 2–10. The total surface area (tPSA) due to polar atoms of a molecule is used to determine the drug transport parameters [35] and compounds with a smaller tPSA value have greater drug transport potential. The tPSA values for the synthesized compounds are as follows: **HL** (37.00 Å^2^) and **1**-**2** (73.21 Å^2^). The **HL** and complexes follow the Lipinski Rule of five (MW < 500, LogP < 5, No. H-bond acceptors < 10, No. H-bond donor < 5) [35]. Figure 8 represents the bioavailability radar image of **HL** and complex **1**.

Drug likeness of a compound indicates the resemblance between the compound and effective drugs [36,37], and it is determined from its Fsp^3^ value which corresponds to the percentage of sp^3^-carbon atoms in the molecule. About 84% of commercially available drugs have Fsp^3^ ≥ 0.42 [38]. For the synthesized compounds, the Fsp^3^ values are 0.12 (**HL**) and 0.14 (**1**, **2**). The molar refractivity values for the screened compounds are 37.90 (**HL**) and 102.50 (**1**, **2**). The predicted octanol/water partition coefficients (logP_o/w_) were in the range of 0.00−6.15.

The logP value, a measure of lipophilicity that determines the overall quality of a drug molecule, is of great importance [39,40], and it can be evaluated by various drug-likeness filters, i.e., MLogP for Lipinski, WlogP for Ghose and Egan, and XlogP for Muegge filters, as well as their mean values (consensus logP). The recommended range of lipophilicity (logP) for a drug is from −0.4 to 5.6 [31]. All logP values for the **HL** and compounds **1** and **2** are in the recommended range (except XLOGP and WLOGP in the case of **1** and **2** which are very slightly higher than the limit).

The estimated aqueous solubility (ESOL) of a compound is an important parameter in drug discovery, and the ideal range of the ESOL is from 5 to 8 [41]. The ESOL values for the screened compounds are −2.34 (**HL**) and −13.25 (**1** and **2**), which fall in the class of soluble to insoluble.

A bioavailability score (BS) is expressed as the probability of a compound with >10% bioavailability in rat or Caco-2 permeability [42], and the recommended value is 0.11 for compounds with tPSA > 150 Å^2^, while the recommended value is 0.85 for compounds with tPSA < 75 Å^2^. The BS values for the screened compounds are 0.85 (**HL**) and 0.55 (**1** and **2**).

The human gastrointestinal absorption values were high for the **HL** and the complexes **1** and **2** [43]. P-glycoprotein and cytochromes P450 can be evaluated in terms of pharmacokinetics. The **HL** does not act as the P-glycoprotein substrate which detects that there is no effect for such protein on its bioactivity, while the complexes **1** and **2** act as the P-glycoprotein substrate which shows that there is an effect for such protein on the bioactivity of these compounds.

The value of skin permeation is expressed as log(Kp), and for compounds with a molecular weight (MW) in the range from 10 to >750, the value of log(Kp) is from −3 to +6 cm/s. A high negative value of log(Kp) indicates that the compound has less penetration into the skin [44]. The values of log(Kp) for the screened compounds are −6.01 (**HL**) and −4.98 (**1** and **2**). Thus, the free ligand acid **HL** has greater skin penetration ability than its complexes [45].

Synthetic accessibility of drug-like molecules is needed in many areas of the drug discovery process. Its values for the evaluated compounds (1.54 for **HL** and 4.97 for **1** and **2**) fall in the range of commercially available drugs [46].

## 3. Materials and Methods

Copper(II) chloride dihydrate; 3,4-difluorophenylacetic acid; 2-methylpyridine; 3-methylpyridine; 2,2-diphenyl-1-picrylhydrazyl radical (DPPH); ascorbic acid; iron(II) sulfate; hydrogen peroxide; nitric acid; sodium salt of the salmon sperm DNA (SS-DNA); methanol; and DMSO were purchased from Sigma-Aldrich, St. Louis, MO, USA. These chemicals were used without any further treatment. Distilled water was used for the experimental work. The melting points of the synthesized complexes were recorded in a capillary tube using a digital electro-thermal melting point apparatus. Elemental analysis was performed on a Leco CHNS 932. A Perkin Elmer atomic absorption spectrometer A analyst 700 was used to determine the percentage of copper. The electronic absorption spectra (200–800 nm) of the complexes were recorded using a Perkin Elmer UV/Vis spectrometer Lambda 25 in DMSO solvent. A nicolet-6700 FT-IR spectrophotometer (Thermo Scientific, Waltham, MA, USA) was used to record FT-IR spectra in the range of 4000–400 cm^−1^, adopting the attenuated total reflectance (ATR) technique.

### 3.1. Syntheses of the Complexes

For the synthesis of complexes **1** and **2**, 3,4-difluorophenylacetic acid (3.44 g, 20 mmol) dissolved in methanol (15 mL) was added dropwise to the constantly stirred solution of copper(II) chloride dihydrate (1.70 g, 10 mmol) in methanol (15 mL). The reaction mixture was refluxed for 3 h followed by the addition of 2-methylpyridine (1.94 mL, 20 mmol) for complex **1** and 3-methylpyridine (1.93 mL, 20 mmol) for complex **2**. The resulting reaction mixture was refluxed for a further 3 h. Then, the reaction mixture was cooled to room temperature. The solution was transferred to a beaker and kept undisturbed. Sea green color crystals were obtained after a few days, and they were separated by filtration and analyzed by different analytical and spectroscopic methods.

**[Cu_2_(3,4-F_2_C_6_H_3_CH_2_COO)_4_(2-CH_3_py)_2_] (1)**

Molecular mass: 997.83; Color: Sea green; M.P. 145–146 °C; Yield: 68%. Anal. Calcd. For C_44_H_34_ Cu_2_F_8_N_2_O_8_(%): C, 52.96; H, 3.43; N, 2.81. Found: C, 52.93; H, 3.47; N, 2.79; FT-IR (cm^−1^): 1624 *ν*(COO)_asym_, 1397 *ν*(COO)_sym_, Δ*ν* = 227, 1208 *ν*(Ar-F), 727 pyridyl ring vibration, 475 *ν*(Cu-N), 418 *ν*(Cu-O); AAS Anal. Calcd for Cu(%): 12.74. Found: 12.71. UV-VIS. [MeOH; λ_nm_]: 733 (d-d transition), 272 (charge transfer transition).

**[Cu_2_(3,4-F_2_C_6_H_3_CH_2_COO)_4_(3-CH_3_py)_2_] (2)**

Molecular mass: 997.83; Color: Sea green; M.P. 154–155 °C; Yield: 65%. Anal. Calcd. For C_44_H_34_Cu_2_F_8_N_2_O_8_ (%): C, 52.96; H, 3.43; N, 2.81. Found: C, 52.92; H, 3.46; N, 2.80; FT-IR (cm^−1^): 1615 *ν*(COO)_asym_, 1396 *ν*(COO)_sym_, Δ*ν* = 219, 1208 *ν*(Ar-F), 725 pyridyl ring vibration, 449 *ν*(Cu-N), 432 *ν*(Cu-O); AAS Anal. Calcd for Cu(%): 12.74 Found: 12.66. UV-VIS [MeOH; λ_nm_]: 754 (d-d transition), 263 (charge transfer transition).

### 3.2. Single-Crystal X-ray Analysis

Diffraction data were collected by the ω-scan technique, at 100(1) K, on a Rigaku XCalibur four-circle diffractometer with an Eos CCD detector, equipped with a graphite-monochromatized MoK_α_ radiation source (λ = 0.71073 Å). The data were corrected for Lorentz polarization as well as for absorption effects [47]. The structures were solved with SHELXT [48] and refined with the full-matrix least-squares procedure on F^2^ by SHELXL-2013 [49]. All non-hydrogen atoms were refined anisotropically, hydrogen atoms were placed in idealized positions and refined as ‘riding model’ with isotropic displacement parameters set at 1.2 (1.5 for methyl groups) times the U_eq_ of appropriate carrier atoms. In structure **2**, one of the difluorophenyl rings was disordered over two positions, rotated approximately by 180°. The site occupation factors for these positions converged at 65.8(4)%:34.2(4)%. Weak restraints were applied to the shapes of displacement ellipsoids in disordered fragments.

### 3.3. Hirshfeld Surface Analysis

The X-ray single-crystal cif files were used as input files to generate the Hirshfeld surfaces as well as 2D-fingerprint plots using Crystal Explorer 17.50 [50]. The following default settings were applied: property, none and resolution, high (standard). For fingerprint generation (di vs. de plot) we applied: range = standard and filter = by elements, whereas fingerprint filter options were both in- and outside elements including reciprocal contacts. The interactions with normalized contact distance in crystal structure that are shorter than the sum of the corresponding van der Waals radii of the atoms are shown as red spots, while those with longer contacts having the positive d_norm_ value are highlighted in blue color. White regions correspond to the distance of contacts that are equal to the van der Waals separation having a d_norm_ value of zero [51].

### 3.4. DNA Binding Study

The complexes were screened for their binding potential to salmon sperm DNA (SS-DNA) by using UV-visible absorption spectroscopy and viscometry.

#### 3.4.1. DNA Binding Study by UV-Visible Absorption Spectroscopy

Sodium salt of the SS-DNA (10 mg) was dissolved in distilled water. The solution was continuously stirred for 24 h. The solution was found sufficiently free of protein impurities as indicated by the nucleotide to protein (N/P) ratio of ~1.9, observed at 260 and 280 nm (A_260_/A_280_) [52]. The SS-DNA solution (ε = 6600 M^−1^ cm^−1^ at 260 nm) concentration was determined via absorption spectroscopy [53]. The complex solutions (200 μM) were made in aqueous DMSO (1:4). The absorption titrations were performed with fixed concentrations of the complex solutions (200 μM) against varying concentrations (5–30 μM) of SS-DNA solutions. To eliminate the absorbance of DNA, equivalent SS-DNA solutions were added to the complex and reference solutions. The complex-DNA solutions were incubated for one hour at room temperature before absorption titrations. For the absorption spectra, cuvettes of 1cm path length were used at room temperature (25 ± 1 °C).

#### 3.4.2. DNA Binding Study by Viscometry

The viscosity of the SS-DNA solution (87.72 μM) in the absence and the presence of complexes **1** and **2** (5–30 μM) was measured using an Ostwald viscometer. For accuracy, the readings were made in triplicate for each sample, and then an average flow rate was calculated. Data are presented as (η/η_o_)^1/3^ vs. binding ratio ^®^ of [complex]/[DNA], where η is the relative viscosity of the complex-DNA adduct, and η_ο_ is the relative viscosity of DNA alone. The relative viscosity values for SS-DNA in the presence (η) and the absence (η_ο_) of the complexes were calculated using the relation η = (t − t_o_)/t_o_, where t is the observed flow time in seconds [54].

### 3.5. Antioxidant Activities

#### 3.5.1. DPPH Free Radical Scavenging Activity

The in vitro DPPH free radical scavenging activities of the free ligand acid and complexes **1** and **2** were assessed, as per a reported procedure with slight modification in the protocol [55].

Different concentrations (15, 30, 60, 120, and 240 μM) of the free ligand acid and complexes were mixed with 1000 μL of 255 μM DPPH solution made in ethanol. The mixture solutions were shaken and incubated in the dark for 30 min at room temperature. The absorbance of the solutions was measured at 518 nm using a UV-VIS spectrophotometer. Ascorbic acid of fixed concentration was used as a positive control. The readings were made in triplicate. Percentage inhibition of the test compounds was evaluated by using the formula:

$$\%scavenging=\frac{A_{c}-A_{s}}{A_{c}}\times 100$$

where A_c_ and A_s_ are the observed absorbance values of the control and the sample, respectively.

#### 3.5.2. Hydroxyl Radical Scavenging Activity

The hydroxyl radical scavenging activities of the free ligand acid and synthesized complexes (**1** and **2**) were evaluated according to a reported method [56]. Different concentrations (15, 30, 60, 120, and 240 μM) of the free ligand acid and complexes were mixed with 0.4 M phosphate buffer (pH 6.6) (1000 μL), 0.1% hydrogen peroxide (100 μL), 3.7 mM *o*-phenanthroline (200 μL), and 7.5 mM ferrous sulfate (100 μL). The mixtures were diluted with distilled water to 3000 μL before incubating for 30 min at room temperature. The absorbance of mixtures was recorded at 510 nm using ascorbic acid as a standard.

The %scavenging was calculated using the following formula:

$$\%scavenging=\frac{A_{s}-A_{c}}{A_{b}-A_{s}}\times 100$$

where A_s_, A_c_, and A_b_ are the observed absorbance values of the sample, control, and blank, respectively.

### 3.6. Amylase Enzyme Inhibition Study

A reported protocol with slight modification was used to perform the α-amylase enzyme inhibition assay [57]. A stock solution (1 unit/mL) was prepared by dissolving α-amylase solution from *Saccharomyces cerevisiae* in 50 mM potassium phosphate buffer (pH 6.8), and for the substrate, in the same buffer 20 mM *p*-nitrophenyl-α-D-glucopyranoside (PNG) was prepared. The assay was conducted in triplicate using 96-well plates. Sample solutions (62.5, 125, and 250 μM) were made in DMSO solvent. For the experiments, each well contained phosphate buffer 65 μL (50 mM, pH 6.8), PNG 25 μL, α-amylase enzyme 5 μL (0.05 U/mL), and test sample 5 μL. The plates were allowed to incubate at 37 °C for 30 min, followed by the addition of 100 μL sodium bicarbonate (0.5 mM) as a stopping agent. Acarbose and DMSO were used as positive and negative controls, respectively.

By using a microplate reader (BioTek Elx-800, Winooski, VT, USA), absorbance was recorded at 405 nm. The percent inhibition was calculated using the formula:

$$\%Inhibition=\frac{Acontrol-Asample}{Acontrol}\;\times \;100$$

where A_control_ is the control absorbance and A_sample_ is the sample absorbance.

### 3.7. Molecular Docking Studies

The molecular docking study was performed using the MOE (2016) software. The 3D structure of alpha-amylase with PDB ID (3BAJ) and salmon sperm DNA (1BNA) was retrieved from the protein databank (PDB) database [58,59]. All the water molecules were removed prior to energy minimization. Energy minimization was performed up to 0.05 gradients with MMFF94s force field. The 3D structure coordinates of the free ligand acid, synthesized complexes, and control (acarbose) were built and saved in a new MOE database. The substrate-binding site of alpha-amylase was specified for molecular docking, while the blind dock approach implemented in MOE was used for the screening of test compounds against SS-DNA [60]. The docking score was used as a scoring function to predict the binding free energy and binding affinity of both the ligand and the target, once it was docked. A more negative value is indicative of a high binding affinity. In order to obtain the low-energy compound protein and SS-DNA compound, the synthesized complexes, free ligand acid, and control compound atoms were set to be flexible during docking. The GBVI/WSA dG scoring function was used to rank the complex activity against the corresponding targets [61]. The interactions of the test compounds with SS-DNA were analyzed using the visualization tool of MOE, and for amylase, the interaction analysis was carried out using Pymol v .1.8. [62].

## 4. Conclusions

In the present work, two dinuclear paddlewheel copper(II) complexes of the type [Cu_2_(3,4-F_2_C_6_H_3_CH_2_COO)_4_(L)_2_], where L = 2-methyl pyridine (**1**) and 3-methyl pyridine (**2**) were synthesized and characterized by elemental, UV-VIS, FT-IR, and single-crystal X-ray analyses. The in vitro antioxidant, *α*-amylase enzyme inhibition, and SS-DNA interaction studies were performed via viscometry and UV-VIS absorption measurements. The result showed improved activities for the synthesized complexes compared to the free ligand acid. The enhanced activities of the complexes could be a consequence of the redox active nature and coordination sphere expansion ability of the Cu(II) ion in the complexes. The molecular docking analyses showed significant interactions of the complexes with *α*-amylase enzyme and SS-DNA. Despite a very close structural/composition resemblance, the complexes showed different efficiencies in antioxidant, *α*-amylase enzyme inhibition assays, and DNA interaction studies, which could be considered to be a consequence of the position of the methyl group on the pyridine co-ligand. In silico studies revealed that **HL** had the highest drug score among the tested compounds. These compounds possess high to low gastrointestinal absorption and blood brain brayer (BBB) permeability properties. Good bioavailability and drug scores along with greater skin absorption further suggest these compounds as possible drug candidates.

## Figures and Tables

**Table 1:** Crystal data, data collection, and structure refinement for complexes **1** and **2**.

Compound	1	2
Formula	C_44_H_34_Cu_2_F_8_N_2_O_8_	C_44_H_34_Cu_2_F_8_N_2_O_8_
Formula weight	997.81	Formula weight
Crystal system	monoclinic	triclinic
Space group	P2_1_/n	P-1
a (Å)	12.9459(7)	10.3143(3)
b (Å)	10.6025(5)	14.0425(5)
c (Å)	14.6950(6)	14.9152(5)
α (°)	90	73.596(3)
β (°)	90.556(4)	77.236(3)
γ (°)	90	85.716(3)
V(Å^3^)	2016.92(17)	2020.97(12)
Z	2	2
D_x_ (g cm^−3^)	1.643	1.640
F(000)	1012	1012
µ (mm^−1^)	1.150	1.148
Reflections:		
collected	20,662	38,873
unique (R_int_)	3562 (0.0873)	7928 (0.0421)
with I > 2σ(I)	2549	6278
R(F) [I > 2σ(I)]	0.0513	0.0323
wR(F^2^) [I > 2σ(I)]	0.1210	0.0718
R(F) [all data]	0.0837	0.0496
wR(F^2^) [all data]	0.1334	0.0772
Goodness of fit	1.029	1.041
max/min Δρ (e·Å^−3^)	0.93/−0.38	0.56/−0.38
CCDC number	2129135	2129136

**Table 2:** Relevant geometrical parameters (Å, °) with standard deviations in parentheses.

	1	2A	2B
Cu1-O1A	1.958(3)	1.9667(15)	1.9595(17)
Cu1-O1B	1.971(3)	1.9703(17)	1.9784(16)
Cu1-O2A ^i^	1.965(3)	1.9800(16)	1.9728(17)
Cu1-O2B ^i^	1.969(3)	1.9828(17)	1.9763(16)
Cu1-N1C	2.194(3)	2.1347(19)	2.1432(18)
Cu1⋯Cu1 ^i^	2.6775(9)	2.6348(6)	2.6318(5)
O1A-Cu1-O2A ^i^	166.78(12)	168.10(7)	168.30(7)
O1B-Cu1-O2B ^i^	166.86(12)	167.91(7)	168.19(6)
N1C-Cu1⋯Cu1 ^i^	174.57(10)	170.31(6)	170.34(5)

**Table 3:** DPPH free radical scavenging activity of the free ligand acid **HL** and the complexes (**1** and **2**).

Compound	% Inhibition					IC_50_ (µM) ± SEM
	Dose Conc. (µM)					
	15	30	60	120	240	
**HL**	1.09	3.05	5.07	7.77	12.55	1009.91 ± 1.99
**1**	8.07	15.77	25.39	39.00	51.12	214.30 ± 7.77
**2**	4.045	5.150	14.975	21.500	32.550	364.78 ± 5.31
Vit. C	7.09	15.09	29.98	58.09	82.35	127.35 ± 13.99

**Table 4:** Hydroxyl radical scavenging activity of the free ligand acid **HL** and the complexes (**1** and **2**).

Compound	% Inhibition					IC_50_ (µM) ± SEM
	Dose Conc. (µM)					
	15	30	60	120	240	
**HL**	0.009	1.050	1.070	2.770	6.660	1770.99 ± 1.17
**1**	0.05	10.00	15.04	28.32	39.99	283.14 ± 7.01
**2**	0.04	1.30	14.50	25.00	35.99	307.55 ± 6.89
Vit. C	6.78	10.49	16.87	43.87	70.55	162.23 ± 12.10

**Table 5:** % α-Amylase activity and IC_50_ values of the free ligand acid **HL** and the complexes (**1** and **2**).

Compound	% *α*-Amylase Activity					IC_50_ (µg/mL)
	Concentration (µg/mL)					
	31.25	62.5	125	250	500	
**HL**	1.72	1.9	9.22	19.67	32.95	728.17
**1**	6.03	7.24	22.24	41.12	51.22	437.73
**2**	5.33	8.33	20.46	38.00	50.30	455.32
Acrabose	14.34	26.42	46.39	68.61	84.33	207.72

**Table 6:** Docking score interactions report for the free ligand acid **HL**, the complexes (**1** and **2**), and acarbose, towards *α*-amylase.

Compound	Interaction Residues	Interaction Type	Distance	Energy (Kcal/mol)	Docking Score
**HL**	GLU 233	H-donor	3.01	−2.4	−5.5159
	GLU 233	H-donor	3.04	−1.0	
**1**	GLY 304	H-donor	3.37	−0.8	−7.433
	GLY 304	H-donor	3.39	−0.6	
	HIS 305	C-H–pi	4.00	−0.8	
**2**	ASP 356	H-donor	3.09	−0.5	−6.9598
	HIS 305	H-donor	3.23	−0.7	
	HIS 305	C-H–pi	4.12	−0.9	
Acarbose	THR 163	H-donor	2.88	−1.5	−8.7377
	GLU 233	H-donor	2.93	−0.6	
	GLU 233	H-donor	3.63	−0.8	
	ASP 300	H-donor	3.31	−1.5	
	HIS 201	H-acceptor	3.00	−1.7	

**Table 7:** Docking score interactions report for the free ligand acid **HL** and the complexes (**1** and **2**) against SS-DNA.

Compound	Interaction Type	Interacting Residues	Distance	Energy (Kcal/mol)	Docking Score
**HL**	H-donor	DC 9	3.55	−0.5	−5.1124
**1**	H-donor	DT 7	3.07	−0.5	−6.5914
	C-H–pi	DC 21	3.74	−1.3	
**2**	H-donor	DA 17	3.33	−1.1	−7.4387
	H-donor	DA 17	3.51	−1.1	
	C-H–pi	DA 18	3.40	−0.5	

**Table 8:** In silico properties for **HL** and the complexes (**1** and **2**) determined by SwissADME.

Physicochemical Properties		
	HL	1 and 2
Molecular weight	172.13 g/mol	498.91 g/mol
Fraction Csp^3^	0.12	0.14
No. rotatable bonds	2	5
No. H-bond acceptors	4	8
No. H-bond donors	1	0
Molar Refractivity	37.90	102.50
TPSA	37.30 Å^2^	73.21 Å^2^
**Lipophilicity**		
Log *P*_o/w_ (iLOGP)	1.43	0.00
Log *P*_o/w_ (XLOGP3)	1.89	6.15
Log *P*_o/w_ (WLOGP)	2.43	6.06
Log *P*_o/w_ (MLOGP)	2.52	3.95
Log *P*_o/w_ (SILICOS-IT)	2.39	2.17
Consensus Log *P*_o/w_	2.13	3.67
**Water Solubility**		
Log *S* (ESOL)	−2.34	−6.89
Class	Soluble	Poorly soluble
**Pharmacokinetics**		
GI absorption	High	High
BBB permeant	Yes	No
P-gp substrate	No	No
CYP1A2 inhibitor	No	No
CYP2C19 inhibitor	No	Yes
CYP2C9 inhibitor	No	No
CYP2D6 inhibitor	No	No
CYP3A4 inhibitor	No	No
Log *K*_p_ (skin permeation)	−6.01 cm/s	−4.98 cm/s
**Drug likeness**		
Lipinski	Yes; 0 vio.	Yes; 0 vio.
Ghose	No; 2 vio. MR < 40, #atoms < 20	No; 2 vio. MW > 480, WLOGP > 5.6
Veber	Yes	Yes
Egan	Yes	No; 1 vio. WLOGP > 5.88
Muegge	No; 1 vio. MW < 200	No; 1 vio. XLOGP3 > 5
Bioavailability Score	0.85	0.55
Drug Score	0.49	0.55
**Medicinal Chemistry**		
PAINS	0 alert	0 alert
Brenk	0 alert	0 alert
Leadlikeness	No; 1 vio. MW < 250	No; 2 vio. MW > 350, XLOGP3 > 3.5
Synthetic accessibility	1.54	4.97

## Data Availability

Data is contained within the article and supplementary material.

## Supplementary Materials

The following supporting information can be downloaded at: https://www.mdpi.com/article/10.3390/ph16101462/s1, Figures S1–S8: FTIR spectra of the free ligand acid and complexes **1** and **2**, respectively.
