# Supplementary material for: Designing and Exploration of the Biological Potentials of Novel Centrosymmetric Heteroleptic Copper(II) Carboxylates
Source: Pharmaceuticals (Basel). 2023 Oct 14;16(10):1462. doi: 10.3390/ph16101462 (PMC10610105; doi:10.3390/ph16101462)
Supplement: Supplementary file 1 [file pharmaceuticals-16-01462-s001.zip › pharmaceuticals-2594201-supplementary.pdf]

Supporting Information

Designing and exploration of the biological potentials of novel centrosymmetric heteroleptic Copper(II) carboxylates

Viola [1], Niaz Muhammad *[1], Awal Noor *[2], Muhammad Sirajuddin [3], Maciej Kubicki [4], Shahnaz Rahim [1], Abdus Samad [5], Shaukat Shujah [6], Abdul Wadood [5] and Saqib Ali [7]

[1] Department of Chemistry, Abdul Wali Khan University Mardan, Mardan 23200, Pakistan.
[2] Department of Basic Sciences, Preparatory Year Deanship, King Faisal University, Al-Hassa 31982, Saudi Arabia.
[3] Department of Chemistry, University of Science and Technology Bannu, Bannu 28100, Pakistan.
[4] Faculty of Chemistry, Adam Mickiewicz University, Uniwersytetu Poznanskiego 8, 61-614 Poznań, Poland.
[5] Department of Biochemistry, Abdul Wali Khan University Mardan, Mardan 23200, Pakistan.
[6] Department of Chemistry, Kohat University of Science & Technology, Kohat, 26000 Pakistan.
[7] Department of Chemistry, Quaid-I-Azam University Islamabad, Islamabad 45320, Pakistan.

* Correspondence: drniaz@awkum.edu.pk (N.M.); anoor@kfu.edu.sa (A.N.)

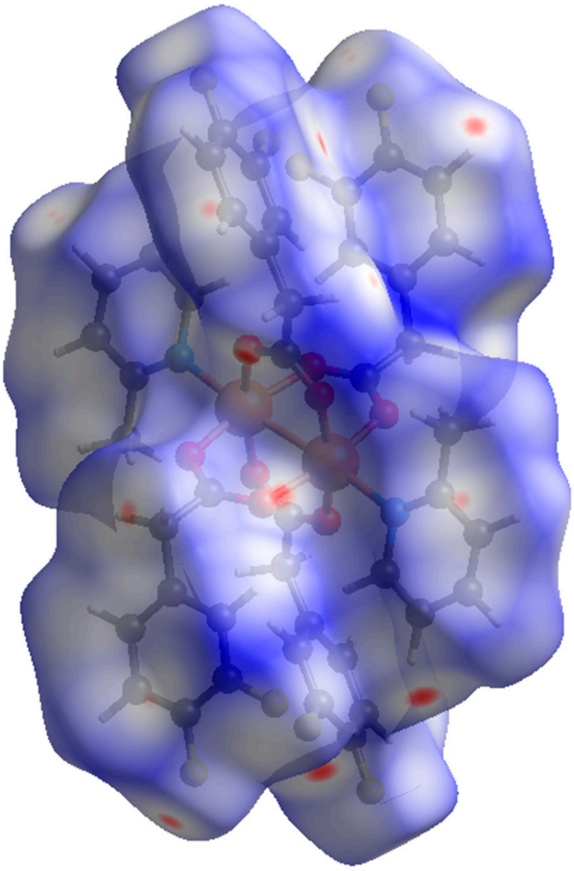

Figure S1. View of the three-dimensional Hirshfeld surface of **1** plotted over d_{norm} in the range −0.1216 to 1.3131.

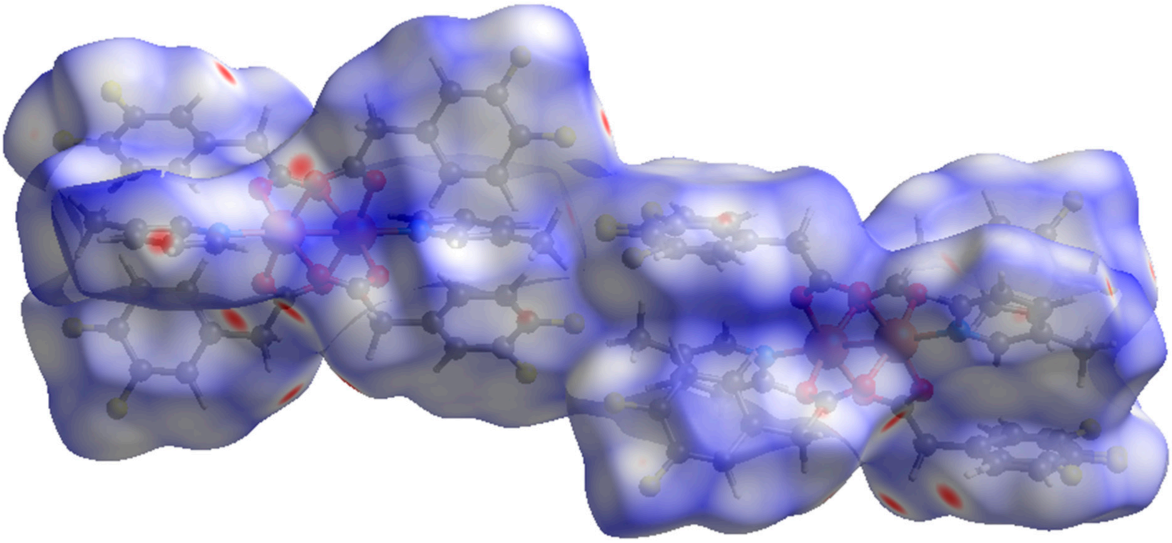

Figure S2. View of the three-dimensional Hirshfeld surface of **2** plotted over d_{norm} in the range −0.1965 to 1.4003.

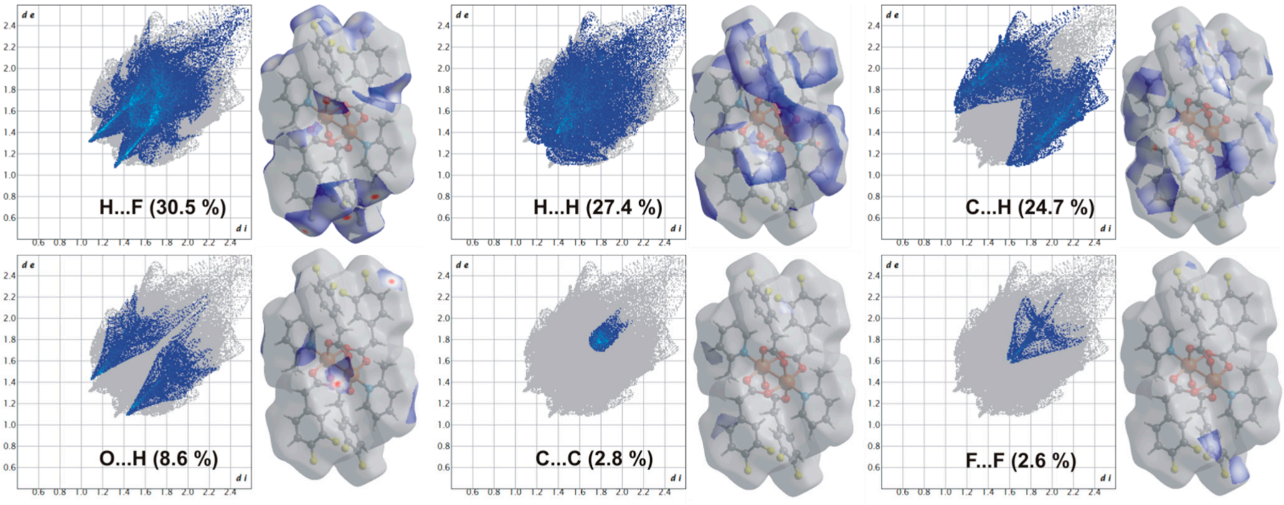

Figure S3. Two-dimensional fingerprint plots for all intermolecular contacts in **1**. The percentage of contribution is specified for each contact.

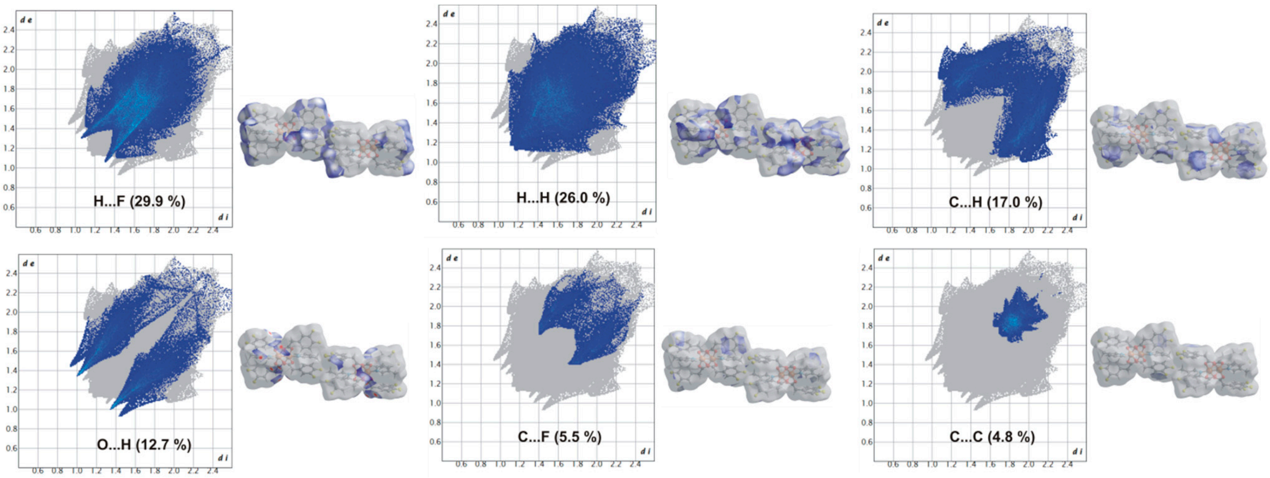

Figure S4. Two-dimensional fingerprint plots for all intermolecular contacts in **2**. The percentage of contribution is specified for each contact.

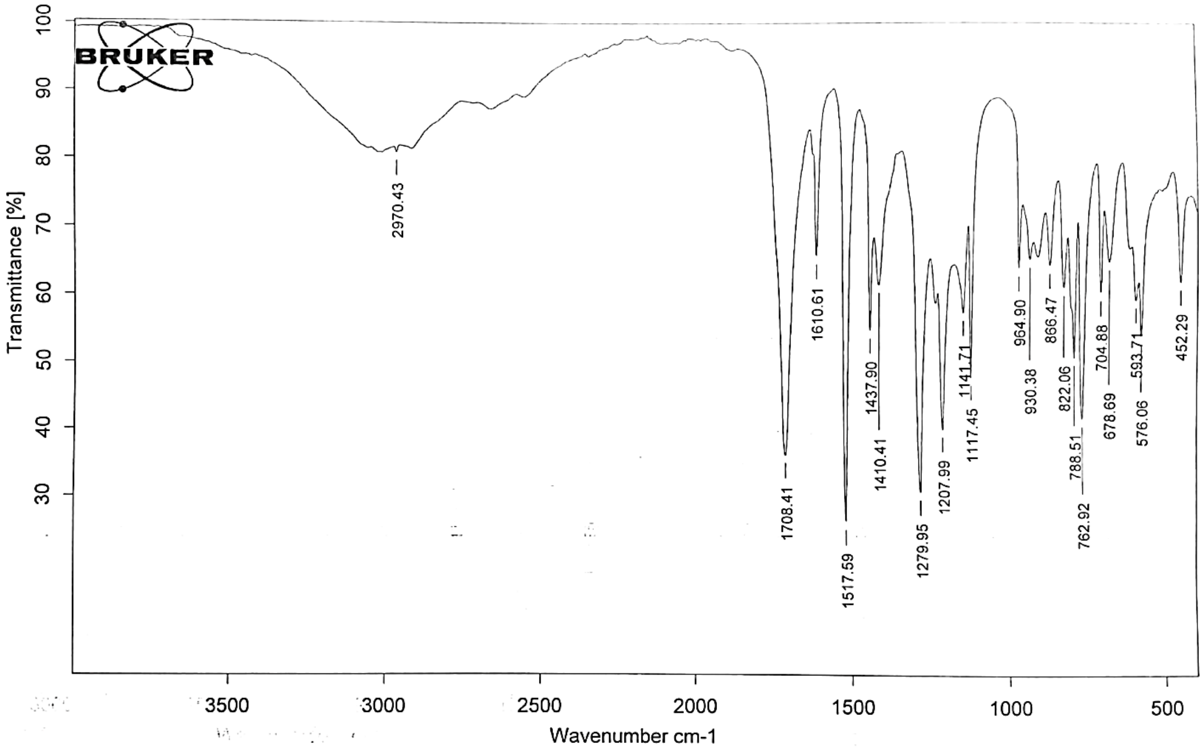

Figure S5: FTIR spectrum of ligand acid.

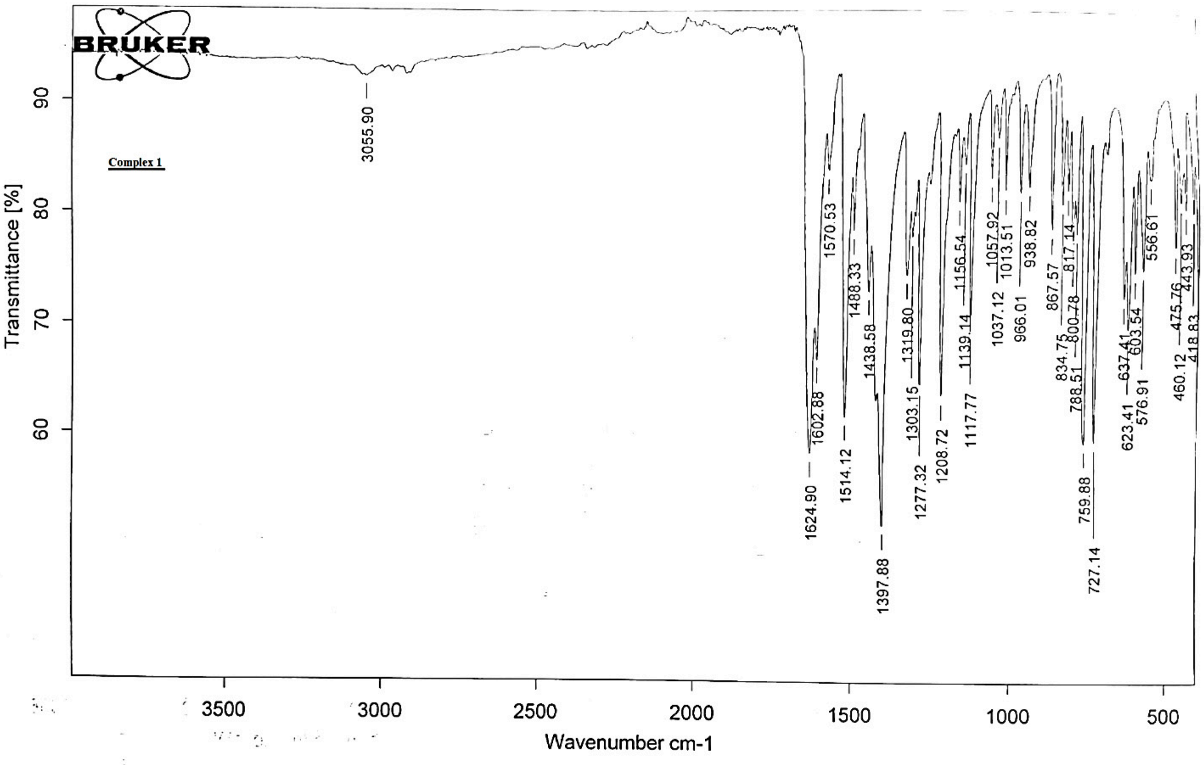

Figure S6: FTIR spectrum of complex **1**.

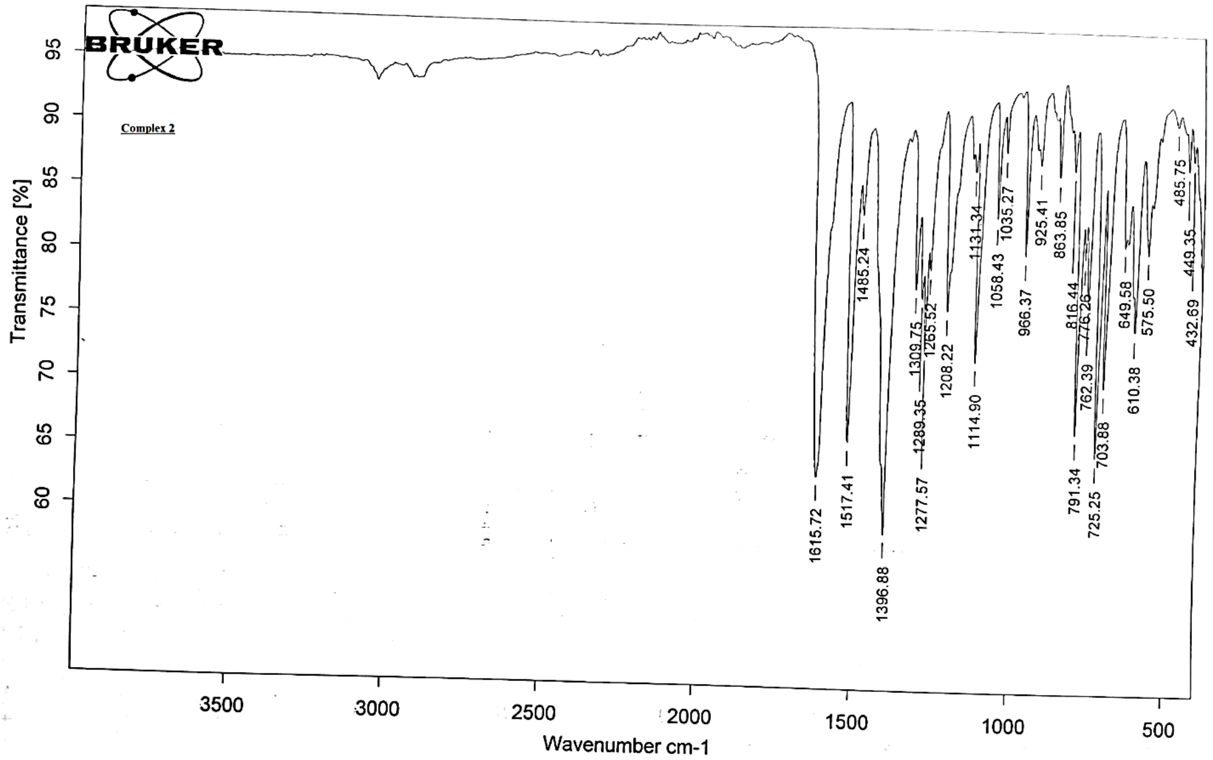

Figure S7: FTIR spectrum of complex **2**.

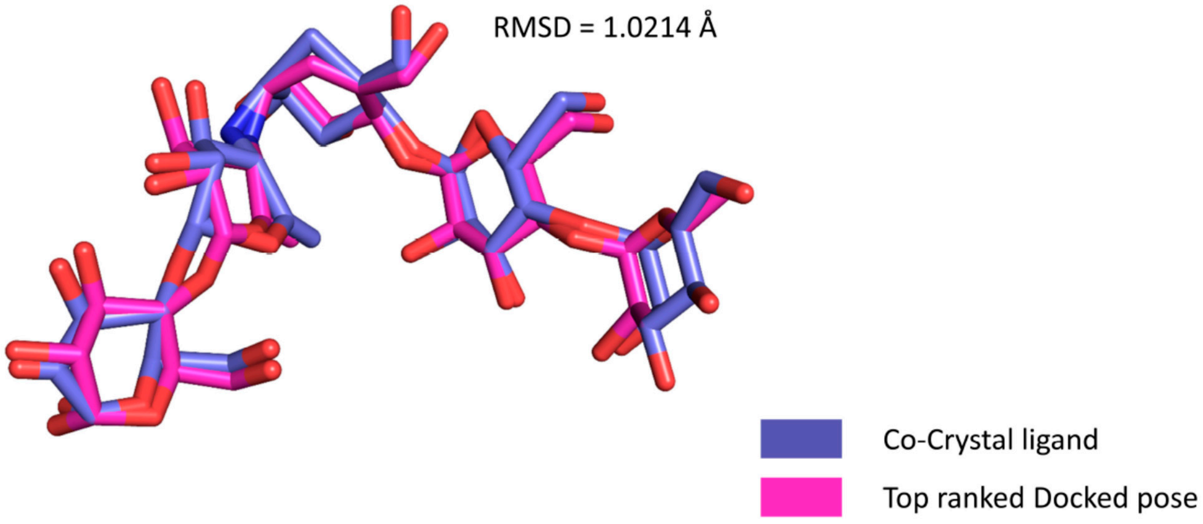

Figure S8: Co-crystal Ligand and Top ranked docked pose.
